# Awareness, Knowledge, and Practices for Preventing Deep Vein Thrombosis in Al Qunfudhah, Saudi Arabia: A Cross-Sectional Study

**DOI:** 10.7759/cureus.66438

**Published:** 2024-08-08

**Authors:** Medhat Taha, Ibrahim Ahmed Aldirhami, Muhannad Hussain Habili, ‏Ismail Abdulmjeed Alkinani, Hashim Hassan Bamusa, Faisal Ali Alhasani, Mohammed Khalid Arishi, Omar Abdullah Alturki, Madani Hussain Habili, Randa M Al Alawi, Nouf Hussain Madani Hibili

**Affiliations:** 1 Department of Anatomy, Umm Al-Qura University, Al Qunfudhah, SAU; 2 College of Medicine and Surgery, Umm Al-Qura University, Al Qunfudhah, SAU; 3 Department of Surgery, South Qunfudah General Hospital, Al Qunfudhah, SAU; 4 Department of Intensive Care Unit, Al Qunfudhah General Hospital, Al Qunfudhah, SAU

**Keywords:** practice., knowledge, awareness, risk factors, venous thromboembolism, deep vein thrombosis

## Abstract

Background

Assessing DVT prevention awareness in the Al Qunfudhah region involves measuring public knowledge about risk factors, prevention, and early treatment through surveys. DVT, characterized by blood clots in the deep veins, poses serious risks, including pulmonary embolism. Raising awareness is crucial for high-risk groups, such as those with prolonged immobility or certain medical conditions, underscoring the need for targeted education and prevention strategies.

Objectives

The objective of the study is to determine the level of awareness regarding deep vein thrombosis (DVT) prevention measures among the general population of Al Qunfudhah governorate.

Methods

This cross-sectional study was conducted in Al Qunfudhah, Saudi Arabia, from January 2024 to April 2024. Data were collected using an online questionnaire targeting individuals aged 18-65 in the region. Analysis was performed using RStudio software version 4.3.1.

Results

Most participants (69.0%) lacked familiarity with DVT. Their knowledge of risk factors, symptoms, prevention strategies, and complications was limited, with a median knowledge score of 8 out of 21 (IQR: 5-11). Familiarity with DVT was an independent predictor of higher knowledge (β = 4.29, 95% CI 3.58-5.00, p < 0.001).

Conclusions

Awareness and knowledge regarding DVT prevention among residents of Al Qunfudhah were found to be inadequate. Targeted educational interventions are needed to improve understanding of DVT and its prevention, especially among those unfamiliar with the condition.

## Introduction

Deep vein thrombosis (DVT) occurs when a blood clot forms in a deep vein, typically in the lower leg or proximal iliofemoral veins. DVT is part of venous thromboembolism (VTE), which also includes pulmonary embolism (PE) [1]. Common symptoms of DVT are pain, tenderness, swelling, redness, and warmth in the affected area [2].

In 1856, Rudolf Virchow introduced Virchow’s triad, identifying three main factors that increase the risk of VTE: venous stasis, endothelial injury, and hypercoagulability [3]. Additional risk factors for DVT include limited mobility, significant surgical procedures, obesity, advancing age, and pregnancy. In surgical patients, factors such as bed rest, vein compression, reduced muscle strength, and a horizontal position contribute to blood flow stagnation [4].

VTE can lead to potentially life-threatening complications, including PE, as well as chronic issues like post-thrombotic syndrome and recurrent DVT. These complications have significant social and economic impacts [4,5]. VTE is a major cardiovascular disease, ranking as the third most common in the Western world. It affects approximately 0.1% of the population annually, with up to 900,000 Americans impacted each year. DVT causes 60,000-100,000 deaths annually in the United States, and in Saudi Arabia, the prevalence is much higher, affecting up to 15.7% of the population. These statistics underscore the urgent need for effective prevention and management strategies [6-8].

The D-dimer assay is a valuable diagnostic tool for DVT, offering high sensitivity but lower specificity. When used with clinical risk assessment, it helps rule out VTE in over 25% of patients with suggestive symptoms [9,10]. Preventing DVT is crucial due to the challenges in detecting and recognizing cases, the substantial costs of managing and treating DVT, and the serious complications that can arise. Effective prevention strategies are essential to address these issues and improve patient outcomes [11,12].

A 2021 study conducted in the Aseer region of Saudi Arabia revealed significant deficiencies in awareness and knowledge about VTE among participants [13]. Raising awareness and assessing knowledge levels are critical initial steps for effective prevention and early detection. Without adequate public awareness, the effectiveness of primary prevention and early detection efforts will be limited, leading to potentially adverse outcomes [14].

The primary objective of this survey is to evaluate the level of awareness, knowledge, and adherence to preventive practices regarding DVT in the Al Qunfudhah area of Saudi Arabia.

## Materials and methods

A descriptive cross-sectional study was conducted targeting residents of the Al Qunfudhah area in Saudi Arabia from January 2024 to April 2024. Adults aged 18 years or older who had lived in Al Qunfudhah for at least six months were included. Children under 18, individuals living outside the Al Qunfudhah governorate, and those who declined to participate were excluded.

An online Arabic questionnaire, designed by the study researchers after an extensive literature review and consultation with field experts, was uploaded to social media platforms via Google Forms. Participants received an electronic link with a brief explanation of the study objectives, the target population, and a request for voluntary participation. After obtaining approval from the Umm Al-Qura University (UQU) Institutional Research Board, the final questionnaire was distributed electronically by data collectors to all eligible individuals.

A convenience sample of 500 adults from the Al Qunfudhah governorate population was randomly selected for data collection. The questionnaire remained available until no additional responses were received.

Study instrument

The scoring method for knowledge of DVT involved evaluating participants’ responses to a set of 10 questions, including four with multiple responses. Each correct answer was scored one point, while incorrect answers received zero points. For multiple-response questions, participants could earn up to 1 point for each correct option selected, with a total possible score ranging from 0 to 21. The questions addressed various aspects of DVT, including its alternative names, seriousness, risk factors, familial predisposition, age demographics, etiology, symptoms, preventive measures, and potential complications.

The attitude score was derived from four items, with one item including multiple responses. These items assessed participants’ willingness to seek medical care for DVT symptoms, familiarity with diagnostic methods, participation in awareness campaigns, and desire for further information about DVT, its symptoms, and treatment strategies. Scores were calculated based on the number of items reflecting positive attitudes, yielding a total score ranging from 0 to 7.

Ethical approval for the study was granted by the Biomedical Research Ethics Committee of UQU, Al Qunfudhah, Saudi Arabia (approval number HAPO-02-K-012-2024-04-2118).

Statistical analysis

Statistical analysis was performed using RStudio software version 4.3.1. Descriptive statistics summarized participants’ demographic characteristics as well as their responses to knowledge and attitude items. Categorical variables were presented as counts and percentages, while continuous variables were summarized using the median and IQR.

The inferential analysis included Wilcoxon rank-sum tests and Kruskal-Wallis rank-sum tests to evaluate differences in knowledge and attitude scores across demographic groups. Multivariable generalized linear regression analysis was used to identify independent factors associated with participants’ knowledge and attitudes toward DVT, with beta coefficients and 95% CIs reported. Statistical significance was set at p < 0.05 for all analyses.

## Results

Demographic characteristics

**Figure 1:**
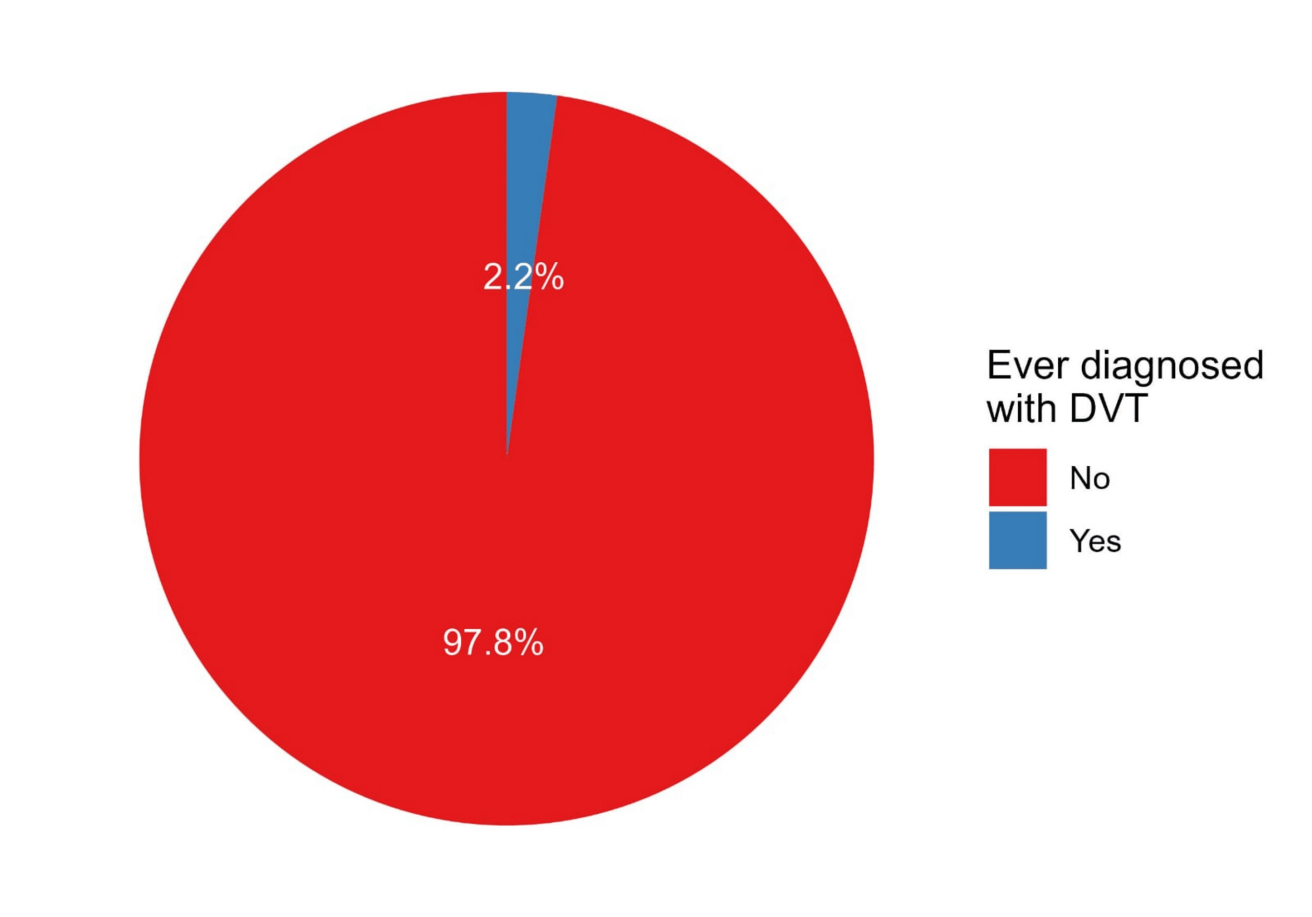
Proportions of participants with a previous history of DVT DVT, deep vein thrombosis

A total of 500 responses were analyzed in this study. Most participants were male (81.2%) and Saudi nationals (99.0%). The largest age group was 18-25 years old (40.0%). University education was the most common among participants, representing 72.9% of the sample. The highest percentage of participants were employed (53.0%). In terms of height, the most common category was 150-169 cm (54.6%), while the most frequent weight category was 70-109 kg (50.6%, Table 1). Eleven participants (2.2%) reported a previous diagnosis of DVT (Figure 1).

**Table 1:** Demographic characteristics

Characteristic	N (%)
Gender	
Male	406 (81.2%)
Female	94 (18.8%)
Age	
18-25	200 (40.0%)
26-35	69 (13.8%)
36-45	121 (24.2%)
46-55	70 (14.0%)
>55	40 (8.0%)
Educational level	
Middle school	4 (0.8%)
High school	83 (16.6%)
Diploma	8 (1.6%)
University	364 (72.9%)
Master’s	28 (5.6%)
PhD	12 (2.4%)
Occupation	
Student	171 (34.2%)
Employed	265 (53.0%)
Unemployed	31 (6.2%)
Retired	33 (6.6%)
Nationality	
Saudi	495 (99.0%)
Non-Saudi	5 (1.0%)
Height (cm)	
<150	14 (2.8%)
150-169	273 (54.6%)
170-190	210 (42.0%)
>190	3 (0.6%)
Weight (kg)	
<50	43 (8.6%)
50-69	188 (37.6%)
70-109	253 (50.6%)
110-140	13 (2.6%)
>140	3 (0.6%)
n (%)	

Awareness and sources of information regarding DVT

A significant proportion of participants (69.0%) reported unfamiliarity with DVT. Among those who were familiar (31.0%), the most common sources of information were the media (40.0%), the educational curriculum (39.4%), and healthcare professionals (32.9%). Fewer participants learned about DVT from someone they knew with the condition (20.0%), and a small percentage (4.5%) had not heard of DVT at all (Table 2).

**Table 2:** Awareness and sources of information regarding DVT DVT, deep vein thrombosis

Characteristic	N (%)
Are you familiar with DVT?	
No	345 (69.0%)
Yes	155 (31.0%)
Sources of information	
Educational curriculum	61 (39.4%)
Healthcare professionals	51 (32.9%)
Media	62 (40.0%)
Someone you know has DVT	31 (20.0%)
I haven’t heard of it	7 (4.5%)
n (%)	

Participants’ responses to knowledge items

In Table 3, participants’ responses to knowledge items about DVT showed that 10.4% correctly identified alternative names for DVT, while 65.2% recognized it as a serious condition. For risk factors, 65.6% identified obesity, whereas oral contraceptive use, prolonged immobilization, smoking, and cancer were recognized by 16.8%, 60.8%, 59.0%, and 19.6% of participants, respectively. Additionally, 43.6% acknowledged a family history of DVT as an important risk factor.

**Table 3:** Participants’ responses to knowledge items DVT, deep vein thrombosis

Characteristic	N (%)
Are there any other names for DVT?	
No	22 (4.4%)
Yes	52 (10.4%)
Do not know	426 (85.2%)
Do you think DVT is a serious condition?	
No	13 (2.6%)
Yes	326 (65.2%)
Do not know	161 (32.2%)
Risk factors for DVT	
Cancer	98 (19.6%)
Oral contraceptive	84 (16.8%)
Obesity	328 (65.6%)
Prolonged immobilization	304 (60.8%)
Smoking	295 (59.0%)
Do you believe that a family history of DVT is an important factor in developing the condition?	
No	61 (12.2%)
Yes	218 (43.6%)
Do not know	221 (44.2%)
Does DVT affect people under the age of 40?	
No	29 (5.8%)
Yes	212 (42.4%)
Do not know	259 (51.8%)
Does DVT occur as a result of stasis of blood (venous stasis), vessel wall injury, and altered blood coagulation?	
No	18 (3.6%)
Yes	249 (49.8%)
Do not know	233 (46.6%)
Symptoms of DVT	
Leg pain	241 (48.2%)
Skin changes on the leg	189 (37.8%)
Swelling in the leg	277 (55.4%)
Visible veins	187 (37.4%)
Are you familiar with any preventive measures or lifestyle changes that can help reduce the risk of developing DVT?	
No	113 (22.6%)
Yes	164 (32.8%)
Do not know	223 (44.6%)
Methods to prevent DVT formation	
Early ambulation after surgeries	178 (35.6%)
Elastic compression stocking	120 (24.0%)
Intermittent pneumatic compression devices	111 (22.2%)
Elevating legs	189 (37.8%)
Foot and leg exercise	355 (71.0%)
Are there any potential complications that can arise from severe or untreated DVT?	
No	27 (5.4%)
Yes	216 (43.2%)
Do not know	257 (51.4%)

Additionally, 42.4% of participants correctly identified that DVT can affect individuals under 40 years old, and 49.8% recognized its etiology, including blood stasis, vessel wall injury, and altered coagulation. Symptoms such as leg pain, swelling, skin changes, and visible veins were correctly identified by 48.2%, 55.4%, 37.8%, and 37.4% of participants, respectively. Moreover, 32.8% were aware of preventive measures or lifestyle changes to reduce DVT risk, and 43.2% recognized potential complications from severe or untreated DVT. Preventive methods like early ambulation after surgeries, leg elevation, and foot and leg exercises were correctly identified by 35.6%, 37.8%, and 71.0% of participants, respectively.

Knowledge score and associated factors

**Figure 2:**
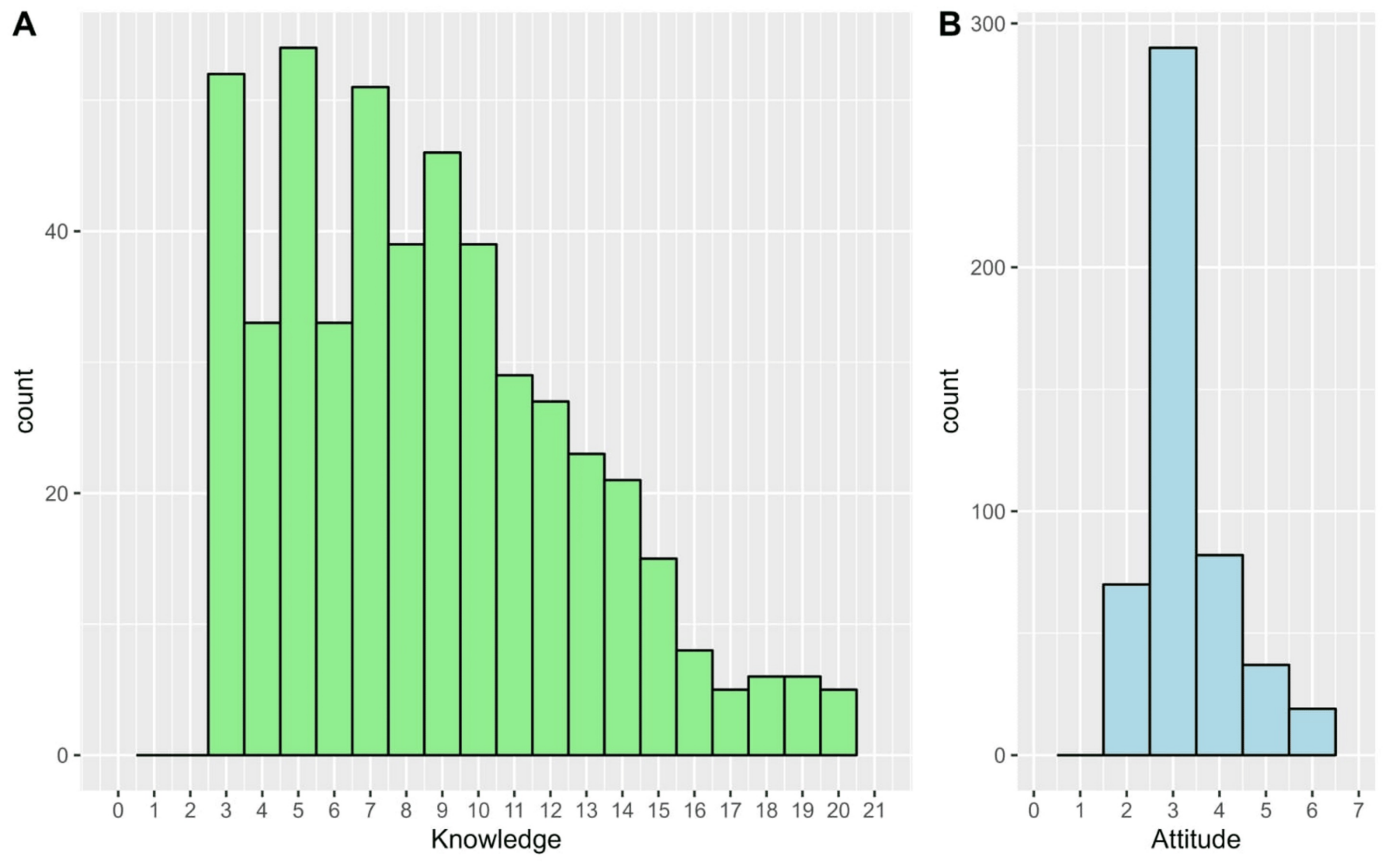
Frequency distributions of the scores of (A) knowledge and (B) attitude

Overall, the median knowledge score was 8.0, with a range from 3.0 to 21.0. The frequency distribution is shown in Figure 2A. Inferential analysis revealed significant differences in knowledge scores based on age (p < 0.001), occupation (p < 0.001), weight (p = 0.004), and familiarity with DVT (p < 0.001). Multivariable regression analysis indicated that participants familiar with DVT had independently higher knowledge scores compared to those unfamiliar with the condition (β = 4.29, 95% CI: 3.58-5.00, p < 0.001, Table 4).

**Table 4:** Factors associated with participants’ knowledge regarding DVT Wilcoxon rank-sum test; Kruskal-Wallis rank-sum test DVT, deep vein thrombosis

Characteristic	Inferential analysis		Multivariable regression		
	Median (IQR)	p-value	Beta	95% CI	p-value
Gender		0.189			
Male	8.0 (5.0, 11.0)				
Female	9.0 (6.0, 12.0)				
Age		<0.001			
18-25	10.0 (7.0, 14.0)		Reference	Reference	
26-35	7.0 (5.0, 10.0)		-0.52	-2.12, 1.09	0.527
36-45	8.0 (5.0, 10.0)		-0.57	-2.18, 1.04	0.485
46-55	7.0 (5.0, 9.8)		-1.12	-2.83, 0.59	0.201
>55	6.5 (3.8, 9.0)		-1.41	-3.62, 0.80	0.211
Educational level		0.175			
Middle school	10.5 (7.8, 12.5)				
High school	7.0 (4.0, 11.0)				
Diploma	7.0 (4.8, 8.5)				
University	8.0 (5.8, 12.0)				
Master’s	7.5 (5.8, 11.0)				
PhD	9.5 (8.0, 12.3)				
Occupation		<0.001			
Student	10.0 (7.0, 14.0)		Reference	Reference	
Employed	7.0 (5.0, 10.0)		-1.25	-2.82, 0.33	0.122
Unemployed	8.0 (5.0, 11.0)		-0.95	-2.48, 0.57	0.22
Retired	7.0 (5.0, 9.0)		-1.93	-4.35, 0.48	0.117
Nationality		0.491			
Saudi	8.0 (5.0, 11.0)				
Non-Saudi	9.0 (5.0, 16.0)				
Height (cm)		0.496			
<150	7.5 (5.3, 11.5)				
150-169	8.0 (5.0, 11.0)				
170-190	8.0 (5.0, 12.0)				
>190	5.0 (4.0, 6.5)				
Weight (kg)		0.004			
<50	9.0 (6.5, 12.0)		Reference	Reference	
50-69	9.0 (6.0, 12.0)		0.09	-1.17, 1.35	0.892
70-109	7.0 (5.0, 10.0)		-0.5	-1.74, 0.74	0.433
110-140	9.0 (6.0, 13.0)		2.1	-0.19, 4.40	0.073
>140	3.0 (3.0, 8.0)		-1.96	-6.31, 2.39	0.378
Ever been diagnosed with DVT?		0.701			
No	8.0 (5.0, 12.0)				
Yes	7.0 (6.5, 8.5)				
Are you familiar with DVT?		<0.001			
No	7.0 (5.0, 10.0)		Reference	Reference	
Yes	11.0 (9.0, 15.0)		4.29	3.58, 5.00	<0.001

Participants’ responses to attitude items

In Table 5, participants’ attitudes toward DVT showed positive trends in several areas. Most indicated a high likelihood of seeking medical care if experiencing DVT-related symptoms, with 44.4% rating their likelihood as 5 out of 5 and 21.8% rating it as 3 out of 5. Regarding diagnostic methods, significant percentages correctly identified CT-angiography (47.2%), MRI (48.0%), and D-dimer (19.6%) as diagnostic tools for DVT, while 27.4% recognized Doppler ultrasound. Despite 91.8% not having participated in DVT-related awareness campaigns, 79.8% expressed a strong interest in receiving more information about DVT, its symptoms, and treatment strategies.

**Table 5:** Participants’ responses to the items of attitudes toward DVT DVT, deep vein thrombosis

Characteristic	N (%)
On a scale of 1 to 5, how likely are you to seek medical care if you experience symptoms related to DVT, where 1 is very low and 5 is very high?	
1	69 (13.8%)
2	35 (7.0%)
3	109 (21.8%)
4	65 (13.0%)
5	222 (44.4%)
Are you familiar with any diagnostic methods or tests used to diagnose DVT?	
CT angiography	236 (47.2%)
D-dimer	98 (19.6%)
MRI	240 (48.0%)
Doppler ultrasound	137 (27.4%)
Have you ever participated in any campaigns or awareness events related to DVT?	
No	459 (91.8%)
Yes	41 (8.2%)
Would you like to receive more information about DVT, their symptoms, and treatment strategies?	
No	101 (20.2%)
Yes	399 (79.8%)
n (%)	

Attitude score and associated factors

The median attitude score was 3.0 (IQR: 3.0-4.0), with the frequency distribution depicted in Figure 2B. Inferential analysis identified significant differences in positive attitudes toward DVT based on age (p = 0.016) and familiarity with DVT (p < 0.001). Multivariable analysis revealed that participants aged 26-35 years were less likely to express positive attitudes compared to those aged 18 to 25 years (β = -0.31, 95% CI: -0.56 to -0.05, p = 0.021). Conversely, participants familiar with DVT demonstrated more positive attitudes than those unfamiliar with the condition (β = 0.35, 95% CI: 0.17-0.53, p < 0.001, Table 6).

**Table 6:** Factors associated with participants’ positive attitudes toward DVT DVT, deep vein thrombosis

Characteristic	Inferential analysis		Multivariable regression		
	Median (IQR)	p-value	Beta	95% CI	p-value
Gender		0.455			
Male	3.00 (3.00, 4.00)				
Female	3.00 (3.00, 4.00)				
Age		0.016			
18-25	3.00 (3.00, 4.00)		Reference	Reference	
26-35	3.00 (3.00, 3.00)		-0.31	-0.56, -0.05	0.021
36-45	3.00 (3.00, 3.00)		-0.1	-0.31, 0.12	0.382
46-55	3.00 (3.00, 3.00)		-0.22	-0.47, 0.04	0.097
>55	3.00 (3.00, 3.00)		-0.34	-0.66, -0.02	0.039
Educational level		0.364			
Middle school	3.00 (3.00, 3.25)				
High school	3.00 (2.50, 3.00)				
Diploma	3.00 (3.00, 4.00)				
University	3.00 (3.00, 4.00)				
Master’s	3.00 (3.00, 4.00)				
PhD	3.00 (3.00, 4.00)				
Occupation		0.105			
Student	3.00 (3.00, 4.00)				
Employed	3.00 (3.00, 3.00)				
Unemployed	3.00 (3.00, 4.00)				
Retired	3.00 (3.00, 3.00)				
Nationality		0.263			
Saudi	3.00 (3.00, 4.00)				
Non-Saudi	4.00 (3.00, 5.00)				
Height (cm)		0.82			
<150	3.00 (3.00, 3.75)				
150-169	3.00 (3.00, 4.00)				
170-190	3.00 (3.00, 4.00)				
>190	3.00 (3.00, 3.00)				
Weight (kg)		0.521			
<50	3.00 (3.00, 4.00)				
50-69	3.00 (3.00, 4.00)				
70-109	3.00 (3.00, 3.00)				
110-140	3.00 (3.00, 3.00)				
>140	3.00 (2.50, 3.50)				
Ever been diagnosed with DVT?		0.902			
No	3.00 (3.00, 4.00)				
Yes	3.00 (3.00, 3.50)				
Are you familiar with DVT?		<0.001			
No	3.00 (3.00, 3.00)		Reference	Reference	
Yes	3.00 (3.00, 4.00)		0.35	0.17, 0.53	<0.001

## Discussion

The current study aimed to evaluate the awareness of DVT prevention among the general population of the Al Qunfudah governorate. Primary prophylaxis, physical measures, and pharmaceutical interventions are key strategies to prevent DVT [1]. Effective nursing interventions can reduce VTE rates in hospitalized patients [2]. Additionally, patient education in healthcare settings is crucial for improving health outcomes [3].

The current study found that over two-thirds of participants were familiar with DVT, primarily through social media, educational curricula, and healthcare professionals. Most people recognize DVT as a serious condition. While about half understood the mechanism of DVT, fewer were aware of the high-risk age group. Reported risk factors included obesity, prolonged immobilization, smoking, cancer, family history, and oral contraceptive use. These findings align with previous studies [4-6].

Symptoms such as leg pain and swelling were known to about half of the participants, with a third aware of skin changes and visible veins. These results are consistent with earlier research [4,5,7], indicating a satisfactory but not high level of knowledge about DVT.

Similar studies showed varying levels of awareness. Alhomayani et al. [8] found that 45.5% of participants had heard of DVT, with long-term travel and being overweight recognized as significant risk factors. Lee et al. [9] reported higher awareness, with 80% of patients and caregivers knowing about DVT, although their risk factors and symptoms differed slightly. In Saudi Arabia, Almodaimegh et al. [10] reported lower awareness, with only 15% knowledgeable about DVT and PE. Alaklabi et al. [11] highlighted that while immobility and old age were recognized as risk factors, awareness of thrombosis as the cause of VTE and its symptoms was less common. A US survey found that 74% of adults had poor knowledge of DVT [12]. Globally, inadequate DVT awareness is a widespread issue, affecting various populations, including pregnant and postnatal women and cancer patients [13-16].

Regarding awareness of preventive measures, the current study found that about one-third of participants were familiar with strategies to reduce DVT risk, while 42% recognized potential complications from severe or untreated DVT. Preventive methods such as foot and leg exercises, elevating legs, and early ambulation after surgeries were the most commonly identified. Yakar et al. [17] reported that 85% of participants administered VTE prophylaxis before surgery, with 76.9% opting for both mechanical and pharmacological prophylaxis concurrently.

In our study, we assessed factors influencing participants’ knowledge about DVT. Age, occupation, weight, and familiarity with DVT were statistically significant in relation to DVT knowledge. Regression analyses revealed that familiarity with DVT was associated with higher knowledge levels. No specific age group, economic status, or educational attainment significantly impacted DVT knowledge in the Al Qunfudhah population. In contrast, Elmahdi et al. [18] found that older age and higher education levels were associated with better VTE knowledge among Saudi adults in Dawadmi. Similarly, Oh et al. [19] reported that stroke awareness was higher among those with higher education. These findings suggest the need for targeted educational initiatives for well-educated individuals to enhance their understanding of DVT symptoms, risk factors, and prevention methods.

The current study revealed that participants had generally positive attitudes toward DVT. Most indicated a high likelihood of seeking medical care if experiencing DVT-related symptoms. While many correctly identified CT angiography and MRI as diagnostic tools, fewer participants recognized D-dimer. Additionally, over three-fourths of participants expressed a desire for more information about DVT, its symptoms, and treatment strategies. Positive attitudes were significantly associated with older age and familiarity with DVT. These findings align with other studies that also report positive attitudes toward DVT, particularly regarding the disease’s severity and the importance of early prevention [8,20,21].

Limitations

This study has several limitations. The online survey required internet access and literacy, potentially excluding segments of the population lacking these resources. The cross-sectional design limits the ability to determine causal relationships. The findings are specific to the Al Qunfudhah governorate and may not be generalizable to other regions or globally. The convenience sampling methodology used could affect sample representativeness compared to random sampling. Despite a large sample size, anonymity and voluntary participation in the survey resulted in self-reported data without verification. Future research with stratified sampling and objective clinical measures could address these limitations and enhance the generalizability of the findings. Overall, while the study offers valuable preliminary insights, further research with improved methodology is needed to confirm the suggested relationships.

## Conclusions

The current study revealed a satisfactory level of awareness and perception toward DVT, particularly regarding risk factors and associated symptoms. Participants also demonstrated considerable awareness of DVT prevention. These findings underscore the need for public education initiatives on DVT. Promoting early diagnosis, treatment, and preventive measures through these initiatives is essential. Further research is needed to identify the most effective methods for raising awareness and disseminating health-related education in Saudi Arabia.
